# Acceptability of a brief fatigue intervention for inflammatory arthritis: a qualitative process evaluation

**DOI:** 10.1093/rap/rkac064

**Published:** 2022-08-16

**Authors:** Alice Berry, Susan Bridgewater, Bryan Abbott, Jo Adams, Emma Dures

**Affiliations:** School of Health and Social Wellbeing, Faculty of Health and Applied Sciences, University of the West of England; Academic Rheumatology, University Hospitals Bristol and Weston NHS Foundation Trust, Bristol; School of Health and Social Wellbeing, Faculty of Health and Applied Sciences, University of the West of England; Academic Rheumatology, University Hospitals Bristol and Weston NHS Foundation Trust, Bristol; Academic Rheumatology, University Hospitals Bristol and Weston NHS Foundation Trust, Bristol; Health Sciences, University of Southampton, Southampton, UK; School of Health and Social Wellbeing, Faculty of Health and Applied Sciences, University of the West of England; Academic Rheumatology, University Hospitals Bristol and Weston NHS Foundation Trust, Bristol

**Keywords:** Fatigue, inflammatory arthritis, cognitive behavioural, process evaluation, acceptability, brief intervention, self-efficacy, rheumatology

## Abstract

**Objectives:**

We developed a brief cognitive behavioural, one-to-one intervention to reduce fatigue impact for patients with inflammatory arthritis. This qualitative process evaluation explored intervention acceptability and potential refinements from the perspective of patients who attended sessions and rheumatology health professionals (RHPs) who delivered the intervention.

**Methods:**

Interviews were conducted with patients and RHPs from five National Health Service (NHS) sites. Data were analysed using inductive thematic analysis.

**Results:**

Twenty-two patients and 11 RHPs participated.

**Patient themes:**

Collaborative, non-judgemental consultations: patients valued having space to reflect, where their fatigue was validated. Relevant content, but not ground-breaking: patients appreciated the opportunity to tailor content to individual priorities. Daily diaries were useful to visualize fatigue. Self-awareness: patients reported increased acceptance, sense of control, and confidence to manage fatigue. Degrees of openness to change: sessions prompted patients to engage in behaviour change. For some, complicated lives made it difficult to plan for change.

**RHP themes:**

Engagement with intervention: RHPs liked training face to face, and sessions were more enjoyable with experience of delivery. Research *vs* clinical practice: RHPs expressed concern about fitting sessions into NHS clinic appointments. It was difficult to offer follow-up sessions within 2 weeks. Collaborating with patients: RHPs reported that patients engaged with the tools and strategies. Some RHPs followed the manual in a linear way, whereas others used it flexibly.

**Conclusion:**

There is potential for this brief fatigue intervention to benefit patients. Future research will focus on flexibility to fit with local services and creating educational resources to use in a range of contexts.

Key messages

- Patients valued the collaborative, therapeutic approach of sessions, describing increased feelings of acceptance and control.
- Rheumatology health professionals liked and enjoyed delivering the sessions, but it was difficult to fit into clinics.
- Potential refinements include developing content that can be applied in varying contexts and formats.

## Introduction

Fatigue is a common, overwhelming and unpredictable symptom in inflammatory arthritis [1, 2], likely to be caused by the complex interaction of clinical factors (e.g. inflammation, pain and disability), psychosocial issues (e.g. coping, mood and behaviours) and personal factors (e.g. working, caring for others and co-morbidities) [3]. UK research with >1200 patients found that 82% wanted support to manage pain and fatigue [4], yet patients report that fatigue is often not addressed in rheumatology consultations [5, 6].

Cognitive behavioural therapy is one of the most helpful types of support for inflammatory arthritis-related fatigue. Previous systematic reviews provide evidence that self-management courses that use cognitive behavioural therapy to increase self-efficacy (beliefs in the ability to carry out self-management behaviours) are more effective than interventions delivering information alone [7–9].

A team from psychology, nursing and occupational therapy designed a brief, low-intensity intervention to address the impact of fatigue on patients with inflammatory arthritis, to be delivered by trained rheumatology health professionals (RHPs), using a manual, over two to four sessions. The first two core sessions were designed to take place face to face and within 2 weeks of each other. Two additional optional sessions could take place face to face or remotely, within the subsequent 4 weeks. It was tested in a feasibility study (Fatigue—Reducing its Effects through individualized support Episodes in Inflammatory Arthritis—FREE-IA) [10].

The intervention aims to reduce the impact of fatigue by encouraging patients to link thoughts, feelings and behaviours influencing their fatigue and their responses to it. Requiring buy-in from both patients and RHPs, the intervention uses an ‘ask, don’t tell’ approach, based on techniques of guided discovery and Socratic questioning [11]. The aim is for patients and RHPs to collaborate and identify relevant lifestyle factors that could be affecting levels of fatigue. The intervention uses tools such as daily activity diaries to assess activity patterns and sets patient-centred goals focusing on changing behaviours. It was designed to be integrated into routine consultations (sessions lasting 20–30 min) and fits well with a stepped approach to care [12] offering a low-resource-intensive treatment option, upon which more intensive services could be added if required.

This paper reports on the findings of a qualitative process evaluation nested within FREE-IA, which played a vital role in understanding the acceptability of the intervention from the perspectives of two groups: the patients who attended sessions and trained RHPs who delivered sessions. It also explored how contextual factors might affect implementation, both as a larger national research study and into normal clinical practice. The aims were to understand the acceptability of the intervention and to identify potential refinements to the intervention.

## Methods

### Recruitment procedures

Six weeks after the first session, the study coordinator telephoned patients to collect their fatigue score. During this call, patients were asked if they would like to take part in an optional telephone interview to discuss their views and experiences. If they agreed, a consent form and information sheet were posted to the patient, and contact details were forwarded to the process evaluation research fellow (A.B.).

The RHPs were recruited via their local principal investigator and were provided with information sheets explaining that they would be invited to take part in an interview. Consenting patients and respective RHPs provided written informed consent by completing the consent form and returning a copy to the study team before interview.

### Data collection and analysis

Two distinct qualitative data sets were collected from patients who participated in FREE-IA sessions and RHPs who undertook FREE-IA training and delivery.

Data were collected via one-to-one interviews by telephone. Interview questions were developed with collaborators and patient partners (Supplementary Data S1, available at *Rheumatology Advances in Practice* online). Patients were asked about the content and acceptability of the intervention. The RHPs were asked about training and the potential for integrating the intervention into clinical practice. Interviews were audio-recorded, transcribed verbatim and anonymized.

An inductive thematic analysis approach [13] was adopted to identify and analyse patterns, without the constraints of fitting data to a predetermined theory. The process evaluation research fellow analysed the transcripts independently, including familiarization with the data by reading the transcripts, initial labelling of early codes to describe small chunks of data that related to the research topic, then starting to search for patterns by grouping together clusters of related codes into initial themes (generally, broader and more abstract than the codes). The study principal investigator contributed to this iterative process by looking at a subset of data to explore what common concepts they saw in the data and how these might be labelled. Both team members then worked together reviewing the themes and sub-themes, regrouping them so that each theme (and related sub-themes) was representative of the data. The study coordinator and patient research partner also reviewed and commented on the themes. The final analysis was agreed in a meeting with the four team members. NVivo (QSR International) was used to organize the data sets.

### Sample

The patient sample was recruited purposively to capture a diverse range of patients, including sex, age range, number of sessions attended, and from across all sites.

All 12 RHPs who took part in FREE-IA intervention training were approached to take part.

## Results

Twenty-two patients took part in an interview. Demographics of the patient sample are presented in Table 1. Eight RHPs agreed to be interviewed, and another three RHPs provided information via email. Interviews took place between March 2019 and May 2020.

**Table 1:** Patient demographics

Characteristic	Patients (*n* = 22)
Sex, *n* (%)	
Male	5 (22.7)
Female	15 (68.2)
Missing	2 (9.1)
Ethnicity, *n* (%)	
White	19 (86.4)
Black	0 (0.0)
Prefer not to say	1 (4.5)
Missing	2 (9.1)
Age, *n* (%), years	
<40	1 (4.5)
40–49	2 (9.1)
50–59	8 (36.4)
60–69	7 (31.8)
70–79	2 (9.1)
Missing	2 (9.1)
Site, *n* (%)	
1 (south-east England)	4
2 (south-east England)	4
3 (south-west England)	7
4 (north-west England)	5
5 (south-west England)	2
Number of sessions attended	
1	3 (13.6)
2	4 (18.2)
3	13 (59.1)
4	2 (9.1)

Findings are presented as two distinct sets: patient interviews and RHP interviews.

### Patient interviews

Four themes capture the views and experiences of the patients and are evidenced using data excerpts. Data were fully anonymized following transcription, and ID codes are used.

#### Theme 1: collaborative, non-judgemental consultations

Patients reported developing positive therapeutic relationships with experienced and knowledgeable RHPs. They felt it was beneficial to have their fatigue validated and to have time and space to reflect on its impact.

I’ve had this for years, and it’s the first time anyone has particularly turned around and said, ‘let’s talk about fatigue’.

(D461)

Just that the fatigue is acknowledged … having a medical professional sit in front of you and say, ‘This is a thing … we understand it’s a thing, we can’t explain why it’s a thing and we can’t give you a tablet to fix it, but we understand it is a thing’.

(D466)

They found the ask don’t tell approach helpful and expressed their preference for a responsive, flexible approach to sessions, rather than a rigid, protocolized approach.

Talking through my specific challenges with a bit of space, and a specialist to give me fresh ideas and not judge me … that one-to-one support and the time to talk about it has been very, very welcome … very, very, helpful.

(D468)

#### Theme 2: relevant and useful, but not ground-breaking

Patients appreciated the range of topics covered and valued the ability of RHPs to tailor content to individual priorities. Some had explored the topics covered previously; however, using visual illustration, for example, to communicate complex issues was very useful and prompted new ways of looking at the issue.

It reinforced really what I should do, and what I needed to think about, and that was helpful. … I mean I knew about trying to get proper sleep and relaxation. Trying to pace oneself, those kinds of things. It’s a case of understanding this, you don’t always remember to do it like that.

(C343)

Being able to visualize the impact of lifestyle patterns on their fatigue using the daily activity diaries was helpful.

You don’t make any real connections, but when you see it … that was a very good visual clue, and I didn’t think that was going to be useful, but actually, I found probably the most useful. It’s such a visual representation of what you are doing or where you are slacking or crashing or whatever it may be.

(D465)

That was the biggest wake-up call for me … looking at the activity diary. Until you look at it—you could see that I didn’t have any pattern or any sort of resemblance to any normality at all. Everything was just chaos.

(B221)

#### Theme 3: increased self-awareness, acceptance and feelings of control

Sessions increased patients’ awareness of lifestyle factors and patterns influencing their fatigue, which increased their sense of control and confidence to manage fatigue.

It just feels like I’ve got more control over fatigue … it’s given me permission and a licence to give myself that care, which I don’t think I was allowing myself before.

(D468)

Some days … take you into nothing but red activity, and today is heading in that direction. I will be ill if I allow that to happen, therefore it’s in my control. I can either do something about it and not feel so bad tomorrow or ignore it and not be able to get up tomorrow.

(B229)

Patients also highlighted how the sessions helped them to accept their fatigue, with this reflective process giving them ‘permission to relax’.

It’s not the be all and the end all now. I accept it is part of the condition, I accept that it might be there more prominent some days than others or some weeks than others. And there’s no point worrying about it.

(A103)

#### Theme 4: degrees of openness to change

Sessions prompted some patients to engage in positive behavioural change, such as adapting sleep patterns, pacing, planning and setting goals.

It started me … paying a bit more attention to things like sleep and diet and lifestyle, but actually specifically thinking about how those could affect the fatigue. … I’m sure the sessions helped with that.

(C344)

For others, the sessions ‘sowed the seeds’ and led to planning for future lifestyle changes.

It’s very easy to get into a rut and just do each day as it comes, and don’t even think about going forward or anything else. … Having things to aim for … I have already booked a few things and doing things in the future.

(D465)

However, some patients felt that any change in lifestyle would not affect their symptoms of fatigue.

I think my condition is governing my fatigue and I don’t think there’s anything that … I think it is what it is, and I think for me the big thing is being more aware of it.

(D461)

For some participants, the broader effects of complex lifestyle situations meant it was difficult to consider or plan for change. Co-morbidities, work and family commitments and lack of finances meant that engaging with positive lifestyle changes was impossible.

### Rheumatology health professionals interviews

Eight RHPs participated in interviews, and three RHPs provided data via email. Three themes capture their views and experiences.

#### Theme 1: engagement with the intervention

The RHPs valued the face-to-face training delivered by the FREE-IA team and learning with peers, describing sessions as providing an opportunity to share ideas for learning.

You get much more engagement when you’re face to face and you’ve got the different dynamics going on in the group … we would discuss the course together and different opinions … I thought it was really good.

(ATTM1)

Some RHPs felt they would have benefitted from refresher training, when starting the sessions was delayed for local logistical reasons.

We all came out all guns blazing (following training), and then … it was a long time before we were able to get on the ground and see people. You kept re-reading the book, but I think it would have been probably better from my idea to have a refresher.

(ETTM1)

For RHPs with extensive experience of providing fatigue support, the low level of treatment intensity and manualized approach limited the usefulness of the intervention. This led to a lower level of engagement and satisfaction compared with RHPs who had fewer resources to use with patients. Conversely, those with less experience of providing fatigue support before the study reported gaining confidence as they delivered more sessions.

As I’ve been doing the sessions, the more confident I’ve got, part of it is knowing the people, but also knowing the material as well. I’ve really enjoyed it, because you can see how much a patient is getting out of it.

(CTTM1)

Yes, ‘ask, don’t tell’—very, very difficult. Because, I mean, by nurses … by definition, we advise our patients on theoretically what we think is best for them … it was quite hard to let go. That was very new. … I think familiarity … the more I did it, the easier it became.

(ETTM1)

#### Theme 2: managing the intervention as a research study and clinical service

Sessions were often carried out outside of usual clinics, or at the end of a clinic in order that more time could be allocated if needed. The RHPs expressed concern about fitting sessions into clinic appointments, because a number of sessions lasted longer than the 20–30 min suggested length. Sessions ranged from 10 to 120 min, with an average (median) of 40 min.

If we were to focus on fatigue alone, no it wouldn’t (work) … if it’s like a five-minute discussion on fatigue and how to manage it, then that’s fine … but anything longer than that … we wouldn’t fit it in.

(BTTM1)

It was a challenge to offer patients a follow-up session within the desired 2-week time frame, because of long waiting times and a high demand for available clinic appointments.

That was quite difficult because of the waiting list I have. Getting them in the first time was all right, but getting them in for the second appointment within a fortnight was quite difficult.

(ETTM2)

Some RHPs described integrating the intervention approach and materials into their routine interactions with patients.

I have already taken advice from the manual and repeated it to patients in clinic. Snippets of useful information is a quick and easy way of helping patients when I am more pressed for time in a ‘normal’ clinic setting.

(DTTM1)

#### Theme 3: collaborating with patients to address fatigue

The RHPs reported that patients were willing to try the tools and strategies during the sessions. Some RHPs followed the manual in a linear way, whereas others adapted content and used it more flexibly.

Obviously, the activity diaries … I think they look at it, not realizing the actual impact it has, once they’ve done it … they don’t realize until they do it.

(CTTM1)

The RHPs also reflected on those patients who did not engage, expressing that some might require a higher-intensity approach and level of support.

Most of the ones who contributed to the study are proactive and want to change, and they are willing to make changes. And then you have got other patients … who think that we can fix them by giving them a tablet, and we can’t. And they put up obstacles about everything you say … but I understand it’s hard…

(BTTM1)

## Discussion

### Acceptability

A key finding within this study was the value of the collaborative, ‘ask, don’t tell’ approach adopted in sessions. Having space and time to discuss the impact of fatigue and having it validated by a health-care professional was empowering. Patients and RHPs described how this approach enabled them to develop positive therapeutic relationships. Some RHPs found using the approach challenging, and this is where more training or experience of the intervention could have strengthened skills and confidence levels of the RHPs.

These findings align with previous literature that highlights the importance of shared decision-making and collaborative working relationships [14]. They strengthen the argument that collaborative, non-didactic consultations are able to foster increased self-efficacy, acceptance of fatigue, confidence in self-management and feelings of control [14–16].

Patients and RHPs highlighted the value of particular tools and approaches, such as using the daily diaries to visualize lifestyle patterns. It is important to recognize elements of the intervention that might be incorporated more easily than others. Adapting the daily diary tool for use in everyday clinics could be explored in future research.

The RHPs became more confident about the (often new to them) interventional approach and content as they delivered more sessions. These findings support earlier fatigue studies [17] and literature reporting a positive relationship between health professional self-efficacy and patient outcomes [18], highlighting the importance that a positive training experience and on-going support might have on health professional self-efficacy and, in turn, patient outcomes.

### Refinements

Patients valued working with a health professional who was familiar to them. The RHPs were able to deliver the sessions, but reported barriers to implementing the intervention into normal practice (in its current format) owing to short clinic appointment slots and the inability to carry out the second session within 2 weeks. Some RHPs had adopted certain tools and topics, using them in everyday clinic appointments. This is an important finding and highlights the possibility of adapting intervention content to make it more acceptable and exploring how it could be incorporated better into a clinical setting.

For some participants, the broader determinants of health impacting levels of fatigue were complex and multidimensional, and factors such as disease activity meant that it simply was not the right time for change. These findings highlight that a low-intensity intervention will not be adequate for all patients experiencing symptoms of fatigue, but recognizing that it has a place within a ‘stepped approach to care’ [12], being beneficial to a proportion of patients, but not an option for all.

### Strengths

This study was able to uncover the experiences and attitudes about the sessions from the perspectives of both the patient and the RHP. It demonstrates sensitivity to context and the wider determinants affecting fatigue, in addition to highlighting the importance of the collaborative approach of the sessions. The methods adopted were able to unpick the workings and nature of the sessions, including issues affecting approach, content and delivery.

The sample was recruited from across all five NHS sites and included participants who had attended between one and four sessions to gain insight into their reasons for opting to take part in more or less of the intervention. Recruitment occurred throughout the course of the study, enabling the sample to include patients who had sessions with RHPs who had differing levels of experience of delivery. Two patient research partners were involved in the design of the study; this included feedback on patient information sheets and interview questions.

### Limitations

The sample size, although relatively small at 22, was close to half of the total number of 46 patients who attended sessions; however, ethnic minority communities were underrepresented in the sample. We also collected no data on the health literacy levels of the patients, which are known to affect self-management strategies in rheumatology [19]. It is acknowledged that the findings might not reflect those of the wider population of patients with inflammatory arthritis who experience fatigue. It is possible that patients who consented to take part in an interview had a more positive experience of the sessions; however, interviews did take place with patients who attended for only one session, all the way through to those who attended all four sessions.

### Next steps

A sensible next step is to explore options for alternative modes of intervention delivery and, importantly, how the essence of the sessions, including the approach and tools, might best be translated. The move to online consultations since the coronavirus disease 2019 (COVID-19) pandemic in 2020 opens up the opportunity for more patients to engage with RHPs, but also presents challenges in terms of accessing intervention resources and working collaboratively. Digitalized content could make it easier for RHPs to provide guidance and copies of the diaries before the first session, meaning that lifestyle patterns can be explored together in the initial session.

Arguably, online working shows potential for how it might be possible to integrate such sessions into clinical appointments. It would be of value to explore this further with RHPs post-COVID-19, in addition to observing what the ‘new normal’ looks like for different rheumatology clinics nationally.

Given the findings of the importance and value of the collaborative nature of the sessions, future research should explore whether and how this approach translates into the digital consultation, and if it is affected in any way, from the perspectives of both the patient and the RHP. Future research will focus on adopting new ways of integrating sessions and content into potentially digital consultations and developing content that can be used in a range of contexts and formats.

### Conclusion

This study reports on the acceptability of a brief, cognitive behavioural, one-to-one intervention to reduce fatigue impact within the NHS. The study presents novel key issues regarding the usefulness for this new intervention and demonstrates the potential for this intervention to benefit patients. Collaborative, positive therapeutic relationships were able to be established between patients and RHPs within a short period of time, and patients described feeling empowered, more in control and confident to address lifestyle patterns and consider positive behavioural change to improve self-management of their fatigue.

## Supplementary Material

rkac064_Supplementary_Data

## Data Availability

Consent was not provided to make data publicly available.
