# Supplementary material for: Acceptability of a brief fatigue intervention for inflammatory arthritis: a qualitative process evaluation
Source: Rheumatol Adv Pract. 2022 Aug 16;6(2):rkac064. doi: 10.1093/rap/rkac064 (PMC9415192; doi:10.1093/rap/rkac064)
Supplement: rkac064_Supplementary_Data [file rkac064_supplementary_data.docx]

# FREE-IA Process Evaluation Paper

# Supplementary Data S1 – Interview guides

**FREE-IA Patient Interview Guide**

**Your fatigue**

- Before your FREE-IA appointments, what was your fatigue like?
- How did fatigue affect your daily life?
- Why did you decide to take part in the FREE-IA study?

**Content and delivery of the FREE-IA appointments**

- How useful did you find the fatigue appointments overall?
- Which parts of the programme did you find more helpful?
  - Explore tools, topics, approaches
- Which parts of the programme did you find less helpful?
  - Explore tools, topics, approaches

Was there anything that could have been clearer?

- Who delivered your programme? How was this?
- What are views on using telephone/Skype to deliver the optional sessions?
- What did you think about the length of each appointment?
- What did you think about the total number of appointments?

**Acceptability and helpfulness of the FREE-IA appointments**

- Can you describe any challenges that you experienced?
  - Practical, emotional
- Can you describe any benefits that you experienced?
  - Practical, emotional
  - How much do you think the appointments will help with your fatigue?
  - How confident are you that you can complete the tasks described in the appointments such as goal-setting or daily diaries?
  - Do you feel that the potential benefits of the intervention outweigh the amount of time and effort needed to attend the sessions? Was it worth it?
  - How important were the topics covered to you? Was it relevant to you personally?
- What changes would you make to the FREE-IA appointments, and why?
  - Anything that you would add in?
  - Anything that you would take out?

**Impact on your fatigue and your daily life**

- Are there ways in which your fatigue has changed since you took part in the study? If so, please can you give some examples?
- Are there ways in which your feelings have changed? If so, please can give some examples?
- Do you do things differently because of the FREE-IA study? If so, please can you describe this and give examples?
- Which elements/advice do you think you might use in the future?

**RHP Interview schedule**

**Prior to FREE-IA**

Please could you tell me about:

1. Your reason(s) for deciding to take part in the FREE-IA feasibility study
2. Any previous experience of supporting patients with their fatigue
3. Any previous experience of using cognitive-behavioural (CB) techniques
4. Any other relevant experience (e.g. training in motivational interviewing)

**Training**

We would like to hear your thoughts on the two-day training that you did in Bristol:

1. The content (e.g. CB theory, manual content, key messages)
2. The structure (e.g. presentation of evidence base, role play, demonstrations)
3. Your experience (e.g. was it what you expected? More/less challenging?)
4. Would you suggest any changes (e.g. more/less skills practice, theory)?
5. How did you feel about the idea of delivering FREE-IA after completing the two-day training (your confidence, motivation, the perceived importance of the intervention)?

**Recruitment**

1. How did you go about identifying suitable participants? – Who talked to potential participants, what did they say/how did they describe FREE-IA?
2. Did your method of recruitment change as you went through the study – i.e. broader/narrower focus?
3. Do you have any ideas why invited/eligible participants may have declined to take part in the study?
4. Did you notice any particular reasons for non-attendance of sessions? (did patients opt out of optional sessions. Were they always offered/needed? (did they want to use phone/skype?)
5. Did you notice any patterns of co-morbidity in the group of participants?

**Intervention Delivery – Delivering the sessions**

1. Did you find the intervention enjoyable to deliver? Particular aspects?/why?
2. Practical challenges (e.g. time to practice and prepare, rooms/setting)
3. Personal/professional challenges (e.g. learning new skills/information, using a manual)
4. Were there particular aspects of FREE-IA that you liked or did not like/problematic?
5. Did you follow the linear nature of the manual, or not? Reasoning etc.
6. Did you find that you and the patient focused more on particular aspects of FREE-IA (e.g. boom & bust)?
7. Do you think that patients found any of the materials more/less helpful?
8. Would you have liked to have access to clinical supervision during the study? If so, what kind of issues would you have liked to discuss?
9. AUDIO-RECORDINGS – How did you feel about the sessions being recorded as part of the research study? Did this affect the sessions in any way?

**Impact of wider clinical practice**

1. Has taking part in FREE-IA had any impact on your wider clinical practice?
   1. If so, can you give me some examples (e.g. materials or techniques that you find useful)
2. Do you perceive any benefits to patients? / Do you perceive any drawbacks for patients?
3. Did you get a sense that this intervention might work better for some than others?
4. Do you perceive any benefits for your professional development? / Do you perceive any drawbacks for your professional development?
5. Have you chosen to use any of the FREE-IA materials in your normal day-to-day practice?
6. Did the FREE-IA sessions impact on day-to-day clinics in any way?
7. How did you feel about the time needed for FREE-IA – recruitment/preparing for sessions/study admin etc?

**Future research or practice**

Thinking about how we might develop and test FREE-IA in the future:

1. Are you and your wider team likely to support the delivery of FREE-IA in the future?
2. Do you think training needs to be face-to-face? In a group? Would DVDs/online training be useful?
3. How would you feel about teleconferencing as a communication method, or for training updates/refreshers?
4. Would you recommend changes to the manual/intervention? If so, can you describe them?
